# Low injury incidence and excellent return to sport after injuries in beach handball—a cross-sectional survey of 651 athletes

**DOI:** 10.1186/s13102-025-01252-w

**Published:** 2025-08-04

**Authors:** Hannes Degenhardt, Maximilian Hinz, Marco-Christopher Rupp, Benjamin D. Kleim, Romed P. Vieider, Maximilian Weyer, Alexander-Stephan Henze, Andreas B. Imhoff, Sebastian Siebenlist, Yannick J. Ehmann

**Affiliations:** 1 Department of Sports Orthopaedics, TUM University Hospital, Technical University of Munich, Ismaninger Str. 22, Munich, 81675 Germany; 2 Department of Orthopedics, Trauma Surgery and Sports Medicine, Muenchen Klinik Bogenhausen, Munich, Germany; 3 Sports and Rehabilitation Medicine, University Hospital Ulm, Ulm, Germany; 4 Department of Orthopaedics, Technical University of Munich, Ismaninger Str. 22, Munich, Germany; 5 Department of Trauma and Orthopaedic Surgery, Klinikum Freising, Freising, Germany

**Keywords:** Team sports, Athletic injuries, Knee injuries, Shoulder injuries, Return to sport, Injury prevention

## Abstract

**Background:**

To quantify the five-year injury incidence and to identify risk factors and common injury patterns in beach handball athletes. It was hypothesized that there would be a low incidence of injuries with identifiable risk and preventative factors.

**Methods:**

An online survey was conducted among active beach handball athletes from 08–09/2022. Demographics, activity level and data on acute and overuse injuries over the past five years were collected. Risk factor analyses were performed for acute and overuse injuries.

**Results:**

A total of 651 athletes (54% male) were included. No injury was reported by 489 (75%) athletes and 162 (25%) athletes reported at least one injury with a total of 174 injuries (102 acute, 72 overuse). The injury incidence was 53.5 injuries per 1,000 athletes per year. The lower extremity was most commonly affected. Older age, the number of tournaments per year and the number of months playing beach handball per year were risk factors for injury. Playing as shooting specialist reduced the risk. Of the injuries, 14 (8%) were treated surgically. Most athletes returned to full beach handball within two months (acute: 70%; overuse: 80%).

**Conclusion:**

Injury incidence was low among beach handball athletes. Injuries most commonly affected the lower extremity. Older age, number of months playing beach handball and number of tournaments were risk factors for injury. Only 8% of the injuries required surgery. Return to sport outcomes was favorable. This implies a potential for targeted injury prevention strategies and the reassurance of low injury burden in beach handball.

**Level of evidence:**

Level III (retrospective comparative study).

**Supplementary Information:**

The online version contains supplementary material available at 10.1186/s13102-025-01252-w.

## Background

Beach handball is a popular sport during summer and beach sporting events and has gained popularity worldwide. Recently, it has been recognized as an Olympic demonstration sport and stands on the verge of becoming an official Olympic discipline [5].

Distinct from its indoor counterpart, beach handball is characterized by its sandy terrain with a smaller court, smaller team size and unique rules that emphasize non-contact play. A match consists of two 10-min periods, with a “shootout” in the case of a draw after both periods. Notably, goals scored through inflight shots, spin shots, or a goal by a goalkeeper or shooting specialist, defined as a specially designated attacking goalkeeper, are counted double. Additionally, a modified substitution rule grants the attacking team a numerical advantage during play [14].

In contrast to beach handball, various studies have reported on injuries during indoor handball [3, 29, 30]. Common injuries in indoor handball include strains and sprains of muscles and ligaments, particularly in the shoulder, knee, and ankle [10, 17, 29]. Indoor handball athletes may also suffer from overuse injuries, such as tendinopathies or stress fractures as a result of repetitive movements. To minimize the risk of injury, handball athletes are required to participate in appropriate training and warm-up exercises and to use correct techniques during matches [16, 18, 22].

Beach handball shares several common characteristics with indoor handball, like physical contact, sprinting, jumping, and throwing. Previous studies have shown that beach handball injuries are similar to those in indoor handball, but occur less frequently [2, 5, 11, 12, 15]. Moreover, recent literature demonstrated that off-season participation in beach handball was associated with a lower incidence of indoor handball injuries, suggesting a potential protective or conditioning effect [8]. Even though many athletes only play beach handball during indoor handball off-season, regular participation can still lead to overuse injuries [2, 5, 11, 12]. Although these studies have provided an understanding of the prevalence of injuries and injury sites in beach handball, there still exists a paucity of data on specific diagnoses, injury mechanisms and timing, risk factors and return to sport (RTS) after injury [2].

Therefore, the aim of the present study was to quantify the five-year incidence of beach handball injuries, to identify common injury patterns and risk factors as well as rates and time to RTS. It was hypothesized that there would be a low injury and that injuries would not be severe, that injuries would occur predominantly to the lower extremity and that they would be mostly contact injuries. It was further hypothesized that acute injuries would occur more frequently compared to overuse injuries and that there would be an overall high RTS rate.

## Methods

### Survey composition and data collection

A team consisting of orthopaedic surgeons, sports medicine specialists and beach handball coaches and athletes developed a questionnaire consisting of 56 questions (see Additional file 1). After a pilot phase including 10 athletes with beach handball experience, the final version of the questionnaire was administered from August to September 2022 through a web-based online survey platform (SurveyMonkey Europe UC, Dublin, Ireland). The survey was conducted in English language and was shared on social media platforms, such as Reddit, Facebook and Instagram with a focus on national and international handball networks to reach beach handball athletes worldwide. In addition, the survey was shared through direct contacts with coaches, team officials, organizers and other stakeholders involved in national and international beach handball structures. Prior approval was obtained from the Ethics Committee of the Technical University of Munich (reference: 654/20S). The study was conducted according to the Declaration of Helsinki. Consent was obtained from all participants before their participation All data were collected anonymously. Incomplete surveys were excluded from all analyses.

The survey consisted of three sections. First, demographic data (gender, country of residence, age, body mass index [BMI]) were collected. The second section focused on training and competition (e.g., years of playing beach handball, training hours per week, tournament participation, games played per year, competitive level and player position). In the third sections, athletes' injuries were assessed by asking participants to report injuries in the last five years of playing beach handball. Injury was defined as any musculoskeletal condition that required medical attention that occurred during training or competition. Injuries were defined as acute (sudden onset of pain or symptoms, associated with an identifiable traumatic event) or overuse (gradual onset of symptoms, not associated with a specific identifiable traumatic event). Recurrent injuries would have been counted individually if they led to separate episodes of time loss and separately analyzed as “recurrent” in the descriptive statistics. To filter for the most significant acute injuries, the questionnaire collected data only for acute injuries that resulted in at least one week of training interruption. All injuries were self-reported without clinical verification. For each reported injury, athletes were asked to indicate the type of injury (acute vs. overuse), the injury location (head/neck, chest wall/torso/abdomen, spine [below neck], shoulder, elbow/arm, hand/wrist, hip/pelvis/thigh, knee/calf/lower leg, ankle or foot) and mechanism, post-injury treatment and time to return to activity (RTA), RTS, and return to competition (RTC).

### Statistical analysis

Statistical analysis was performed using the SPSS 26.0 software package (IBM-SPSS, New York, USA). Normal distribution was tested using the Kolmogorov–Smirnov test. Continuous data were presented as mean ± standard deviation (SD) following confirmation of normal distribution. Categorical variables were reported as frequencies and percentages. Normally distributed continuous variables were compared using an unpaired two-sample t-test. Non-normally distributed continuous variables and categorical variables between groups were compared using the Mann–Whitney U test. Associations between categorical variables were measured using Pearson’s χ^2^-test. Bonferroni correction and post-hoc test were used for multiple pairwise comparisons. A multivariate logistic regression model was used to identify independent risk factors for injury. Variable selection for the multivariate model was performed utilizing a stepwise backward elimination of non-significant factors, with the presence of an injury was defined as the dependent variable. The level of significance, adjusted odds ratios (OR) and 95% confidence intervals (CIs) were calculated. The level of significance was set at *p* < 0.05.

## Results

### Study population

A total of 1,132 athletes participated in the survey. After excluding incomplete questionnaires, 651 athletes (54% male) from 43 different countries were included for further analysis. The five most represented countries were Germany (*n* = 241), Portugal (*n* = 54), Spain (*n* = 53), France (*n* = 34) and the Netherlands (*n* = 21). No notable patterns or differences were observed based on the country of origin. Data on athletes’ demographics and origin, training and competition divided by sex are presented in Table 1.

**Table 1:** Data on athletes’ demographics as well as training and competition for the total study cohort as well as for each sex

	**All athletes (*n* = 651)**	**Male athletes (*n* = 353)**	**Female athletes (*n* = 298)**	***p*-value**
Age, years	25.3 ± 8.0	26.1 ± 8.6	24.4. ± 7.1	.032*
BMI	23.8 ± 3.0	24.5 ± 3.0	23.0 ± 2.7	< .001*
Years played beach handball, n	7.6 ± 5.4	7.88 ± 5.9	7.2 ± 4.9	> .05
Months playing beach handball per year, n	4.0 ± 2.6	4.0 ± 2.7	3.9 ± 2.6	> .05
Throwing arm, n (%)				
Right	590 (90.6)	318 (90.1)	272 (91.3)	> .05
Left	61 (9.4)	35 (9.9)	26 (8.7)	> .05
Play hours per week, n (%)				
0–3	250 (38.4)	125 (35.4)	125 (41.9)	> .05
3–6	236 (36.3)	117 (33.1)	119 (39.9)	> .05
6–9	112 (17.2)	73 (20.7)	39 (13.1)	> .05
10 +	53 (8.1)	38 (10.8)	15 (5.0)	> .05
Tournaments per year, n (%)				
0–3	284 (43.6)	141 (39.9)	143 (48.0)	> .05
3–6	271 (41.6)	147 (41.6)	124 (41.6)	> .05
6–9	83 (12.7)	55 (15.6)	28 (9.4)	> .05
10 +	13 (2.0)	10 (2.8)	3 (1.0)	> .05
Games per year, n (%)				
0–5	75 (11.5)	32 (9.1)	43 (14.4)	> .05
6–10	76 (11.7)	37 (10.5)	39 (13.1)	> .05
11–15	94 (14.4)	49 (13.9)	45 (15.1)	> .05
16–20	128 (19.7)	64 (18.1)	64 (21.5)	> .05
21–25	102 (15.7)	50 (14.2)	52 (17.4)	> .05
25 +	176 (27.0)	121 (34.4)	55 (18.5)	< .001*
Play level, n (%)				
Amateur	75 (11.5)	31 (8.8)	44 (14.8)	> .05
Competitive	361 (55.5)	181 (51.3)	180 (60.4)	> .05
Semi-professional	202 (31.0)	153 (37.7)	69 (23.2)	< .001*
Professional	13 (2.0)	8 (2.3)	5 (1.7)	> .05
Competition level, n (%)				
Local	64 (9.8)	22 (6.2)	42 (14.1)	< .001*
Regional	88 (13.5)	43 (12.2)	45 (15.1)	> .05
Nationwide	183 (28.1)	90 (25.5)	93 (31.2)	> .05
International	316 (48.5)	198 (56.1)	118 (39.6)	< .001*
Position, n (%)				
Goalkeeper	110 (16.9)	67 (19.0)	43 (14.4)	> .05
Defense	259 (39.8)	137 (38.8)	122 (40.9)	> .05
Shooting specialist	204 (31.3)	102 (28.9)	102 (34.2)	> .05
Backfield	48 (7.4)	25 (7.1)	23 (7.7)	> .05
Left wing	202 (31.0)	108 (30.6)	94 (31.5)	> .05
Right wing	156 (24.0)	89 (25.2)	67 (22.5)	> .05
Pivot	133 (20.4)	79 (22.1)	55 (18.5)	> .05

Continuous variables are shown as mean ± standard deviation, categorical variables are shown as number of patients and percentages per group. Bolded *p*-values and asterisks indicates significant difference between groups (*p* < .05)

### Injury incidence, type of injury and injury location

Within the five-year time period of the survey, 489 athletes (75%) reported no injury, 151 athletes (23%) reported one injury, 10 athletes (2%) reported two injuries, and one athlete (0.2%) reported three injuries. A total of 174 injuries were reported, resulting in an overall incidence rate of 53.5 injuries per 1,000 athletes per year (95% CI 45.5–61.4). For male athletes, the injury incidence rate was 53.8 injuries per 1,000 athletes per year (95% CI 45.0–64.7) and for female athletes 53.0 injuries per 1,000 athletes per year (95% CI 41.3–64.7)with no statistically significant difference (*p* > 0.05). Regarding match exposure, the overall injury rate was 9.0 per 1,000 match hours (95% CI 7.7–10.3), with 8.5 in men 95% (CI 6.8–10.2) and 9.7 in women (95% CI 7.6–11.8), with no statistically significant difference (*p* > 0.05). Comparisons between athletes with and without injuries regarding demographic, training and competition data are provided in Table 2. Of all 174 reported injuries, 102 (59%) were acute and 72 (41%) were overuse. Injury locations and frequencies are listed in Table 3.

**Table 2:** Comparison between injured and non-injured athletes with regards to demographic, training and competition data

	**Injured athletes** **(*n*** **= 162)**	**Non-injured athletes** **(*n*** **= 489)**	***p*-value**
Sex, n (%)			
Male	88 (54.3)	265 (54.2)	> .05
Female	74 (45.7)	224 (45.8)	> .05
Age, years	26.1 ±7.2	25.0 ±8.3	.026*
Height, cm	179.4 ±10.9	179.0 ±10.0	> .05
Weight, kg	77.3 ±15.5	76.7 ±14.0	> .05
BMI	23.9 ±3.1	23.8 ±3.0	> .05
Years played beach handball, n	7.9 ±4.5	7.4 ±5.7	.033*
Months playing beach handball per year, n	4.7 ±2.8	3.7 ±2.5	<.001*
Throwing arm, n (%)			
Right	147 (90.7)	443 (90.6)	> .05
Left	15 (9.3)	46 (9.4)	> .05
Play hours per week, n (%)			
0-3	42 (25.9)	208 (42.5)	<.001*
3-6	70 (43.2)	166 (33.9)	> .05
6-9	35 (21.6)	77 (15.7)	> .05
10+	15 (9.3)	38 (7.8)	> .05
Tournaments per year, n (%)			
0-3	52 (32.1)	232 (47.4)	<.001*
3-6	75 (46.3)	196 (40.1)	> .05
6-9	30 (18.5)	53 (10.8)	> .05
10+	5 (3.1)	8 (1.6)	> .05
Games per year, n (%)			
0-5	10 (6.2)	65 (13.3)	> .05
6-10	17 (10.5)	59 (12.1)	> .05
11-15	24 (14.8)	70 (14.3)	> .05
16-20	27 (16.7)	101 (20.7)	> .05
21-25	26 (16.0)	76 (15.5)	> .05
25+	58 (35.8)	118 (24.1)	<.001*
Play level, n (%)			
Amateur	9 (5.6)	66 (13.5)	> .05
Competitive	80 (49.4)	281 (57.5)	> .05
Semiprofessional	70 (43.2)	132 (27.0)	<.001*
Professional	3 (1.9)	10 (2.0)	> .05
Competition level, n (%)			
Local	10 (6.2)	54 (11.0)	> .05
Regional	11 (6.8)	77 (15.7)	<.001*
Nationwide	42 (25.9)	141 (28.8)	> .05
International	99 (61.1)	217 (44.4)	<.001*
Position, n (%)			
Goalkeeper	29 (17.9)	81 (16.6)	> .05
Defense	77 (47.5)	182 (37.2)	.023*
Shooting specialist	36 (22.2)	168 (34.4)	.002*
Backfield	11 (6.8)	37 (7.6)	> .05
Left wing	52 (32.1)	150 (30.7)	> .05
Right wing	43 (26.5)	113 (23.1)	> .05
Pivot	42 (25.9)	91 (18.6)	> .05

Continuous variables are shown as mean ± standard deviation, categorical variables are shown as number of patients and percentages per group. Bolded *p*-values and asterisks indicates significant difference between groups (*p*< .05)

**Table 3:** Injury locations of acute and overuse injuries

Injury location	Acute (*n* = 102)	Overuse (*n* = 72)
Head/neck, n (%)	10 (9.8)	2 (2.8)
Chest wall/torso/abdomen, n (%)	1 (1.0)	0 (0.0)
Spine (below neck), n (%)	2 (2.0)	11 (15.3)
Shoulder, n (%)	14 (13.7)	9 (12.5)
Elbow/arm, n (%)	3 (2.9)	5 (6.9)
Hand/wrist, n (%)	11 (10.8)	4 (5.6)
Hip/pelvis/thigh, n (%)	4 (3.9)	6 (8.3)
Knee/calf/lower leg, n (%)	25 (24.5)	20 (27.8)
Ankle or foot, n (%)	32 (31.4)	15 (20.8)

Categorical variables are shown as number of athletes and percentages per group

The body regions that were most commonly affected by acute injuries were the lower extremity (60%, *n* = 61) and the upper extremity (28%, *n* = 28). The five most common diagnoses of acute injuries were nine fractured toes (9%), six lateral ankle ligament tears (6%), five unspecified ankle injuries (5%), five calf muscle injuries (5%) and five shoulder dislocations (5%), of which three athletes (60%) experienced a dislocation in the setting of recurrent shoulder instability. Fractures were recorded in 19 athletes (19%) of whom one required surgery for a facial fracture. Muscle and tendon injuries occurred in 13 athletes (13%). Concussions were reported by 3 athletes (3%). There were no significant differences regarding the body region and specific diagnosis between male and female athletes with regards to acute injuries. Diagnoses of acute injuries are reported in Additional file 2.

Regarding overuse injuries, the body regions that were most commonly affected were the lower extremity (57%, *n* = 41) followed by the upper extremity (25%, *n* = 18), specifically knee/calf/lower leg (28%, *n* = 20), ankle or foot (21%, *n* = 15) and shoulder (13%, *n* = 9). The spine was affected in 15% (*n* = 11) of all overuse injuries. The five most common diagnosis of overuse injuries were patellar tendinopathy (11%, *n* = 8), achilles tendinopathy (6%, *n* = 4), stress fractures of the foot (6%, *n* = 4), shoulder bursitis (6%, *n* = 4), overuse muscular back pain (6%, *n* = 4) and unspecified knee injuries (6%, *n* = 4). In 50% (*n* = 36) of overuse injuries, injuries were noted to be recurrent injuries. In this cohort, recurrent injuries did not lead to multiple distinct episodes of time-loss. No significant differences regarding the body region and specific diagnosis were observed between male and female athletes. Diagnoses of overuse injuries are reported in Additional file 3.

### Risk factors

The number of months of playing beach handball per year was independently associated with an 11% increase in the odds for any injury (OR 1.11; [95% CI 1.04–1.19]; *p* = 0.002), see Additional file 4. Regarding acute injuries, older age increased injury odds by 5% per year (OR 1.05; [95% CI 1.01–1.09]; *p* = 0.008) and each additional tournaments played per year was associated with a 53% higher odds (OR 1.53; [95% CI 1.09–2.15]; *p* = 0.015), see Additional file 5. For overuse injuries, every additional month playing beach handball per year increased injury odds by 14% (OR 1.14; [95% CI 1.04–1.24]; *p* = 0.005). Playing as the shooting specialist was associated with 55% lower odds of sustaining an overuse injury (OR 0.45; [95% CI 0.23–0.91]; *p* = 0.027), see Additional file 6.

### Timing and mechanism of acute injuries

Of all acute injuries, 66 (65%) occurred during competition and 36 (35%) occurred during training. Among competition injuries, 30 (46%) were sustained in the first half of the game, 28 (42%) were sustained during the second half of the game and 2 injuries (3%) each occurred during warm-up or shoot-out (4 cannot remember [6%]). Of the training injuries, 4 injuries (11%) occurred during the first 15 min of training, 14 injuries (39%) were sustained between the first and last 15 min of training, 9 injuries (25%) were sustained in the last 15 min of training and 2 injuries (6%) occurred during warm-up (7 cannot remember [19%]). Of all acute injuries, 44 injuries (43%) each occurred in offense or defense. There were no significant differences between male and female athletes regarding injury timing. Further details on the timing and injury location on the field of acute injuries are reported in Additional file 7. Of all acute injuries, the majority of injuries (55%, *n* = 56) were caused by contact with either an opponent (46%, *n* = 47) or a team mate (9%, *n* = 9) and 23 injuries (23%) were sustained while landing on the ground. A total of 10 injuries (10%) occurred during a spin shot. There were no significant differences between male and female athletes regarding injury mechanism. Mechanisms of acute injuries are reported in Additional file 8.

### Treatment of acute and overuse injuries

Following acute injury, 26 athletes (26%) were taken to hospital, 66 athletes (65%) received no further treatment, 60 athletes (59%) opted for physical therapy and 8 athletes (8%) underwent surgery (1 × toe injury, 3 × meniscal tear, 1 × lateral ligament tear of the ankle, 1 × dislocated shoulder, 1 × broken bone of the face/skull, 1 × acromioclavicular joint dislocation), see Additional file 9.

After overuse injury, 27 athletes (38%) took a break from training, games and competitions, 29 athletes (40%) underwent physical therapy and 6 athletes (8%) were treated surgically (2 × overuse spondylolisthesis [instability between vertebrae, possibly caused by hyperextension], 1 × patellar tendinopathy, 1 × broken nose, 1 × meniscal tear, 1 × unspecified knee injury), see Additional file 10.

### Return to sport

Of all athletes with acute injuries (*n* = 102), 95 athletes (93%), were able to return to beach handball. The remaining seven athletes were recently injured, of which five athletes were injured between one and three months ago and the remaining two athletes were injured less than one month ago, 70% (*n* = 66) of athletes returned to training or competition and 54% (*n* = 51) returned to the same physical level as before within two months, see Additional file 11.

Of all athletes with overuse injuries (*n* = 72), 66 athletes (92%) were able to return to beach handball. For beach handball, 80% athletes (*n* = 53) returned to training or competition within two months and 60% (*n* = 40) returned to the same physical level as before, see Additional file 12.

## Discussion

### Key findings

This study has revealed several relevant findings in relation to injuries among beach handball athletes. First, the overall incidence of injury in beach handball was low, with only approximately one in four athletes sustaining an injury in the last five years. Second, despite the nature of beach handball as a throwing sport, only 14% of athletes suffered acute shoulder injuries. Thirdly, 160 injuries (92%) were treated non-operatively. Finally, the injury rate in beach handball was lower than in indoor handball. Beach handball injuries rarely necessitated surgery and return to sport rates were over 90% with more than 50% returning to pre-injury levels within 2 months.

### Injury incidence, location and type of injury

The majority of athletes (75%) did not sustain any injury and the injury incidence rate was low (53.5 injuries per 1000 athletes and year; 9.0 injuries per 1,000 match hours). However, as discussed in the limitations section the study setup might have inclined the participants without an injury to complete the questionnaire more often. In comparison to indoor handball, where time-loss injury incidence rates up to 50 injuries per 1000 match hours have been reported, the overall injury incidence rate in beach handball appears relatively low [2, 3, 9, 27, 28]. In beach handball, incidence rates of 11–12 injuries per 1000 h during games and 2–4 injuries per 1000 h during training were reported previously [11]. Furthermore, Achenbach et al. investigated an injury incidence in 30 national teams participating in the 2017 European Beach Handball Championship and found an injury incidence of 49.3 per 1000 match hours although there were no injuries with a time loss of more than seven days [2]. The lower injury incidence observed in beach handball versus indoor handball may be due to multiple reasons. First, soft, forgiving sand surface may result in slower movements. This effect is pronounced during change of direction and may results in a softer impact. Different jumping and throwing techniques, along with the non-contact and fair play rules, may lower the injury risk in beach handball. with potentially offering a protective effect by improving coordination, proprioception, and speed [7, 23]. Furthermore, beach handball participation in the off season helps to maintain fitness and endurance with sport-specific training, reducing musculoskeletal stress while providing equivalent cardiovascular training benefits [4, 6, 13, 24].

Of the reported injuries, 102 were classified as acute with a sudden onset of pain or symptoms, whereas 72 were categorized as overuse. Existing studies confirm this pattern reporting a comparable distribution between acute and overuse injuries in indoor handball [3, 18, 30]. The differentiation between acute and overuse injuries offered valuable insight into injury prevention strategies. Acute injuries may be related to specific game situations or actions, while overuse injuries might result from overuse or repetitive movements.

Notably, acute injuries frequently affected the ankle and foot, followed by injuries to the knee/calf/lower leg. Initially, ankle and foot injuries were combined for analysis. When considering ankle injuries alone, there were 15 ankle injuries (15%), confirming that the pattern of more knee than ankle injuries is consistent with indoor handball [17, 30]. In contrast to the existing literature in indoor handball and the high number of ruptures of the anterior cruciate ligament (ACL), only one ACL strain (0.02%) and no ACL tear was reported in the present study [17]. Furthermore, the increased rate of ACL injuries in women provided by the literature could not be confirmed since no significant gender-specific difference was found in the present study [17, 20, 32]. The existing studies on indoor handball and beach handball show comparable rates for injury localization [2, 3]. In studies comparing beach volleyball with indoor volleyball and beach soccer with soccer or futsal, it was shown that the variant played on sand resulted in fewer ankle injuries [1, 26]. Interestingly, although beach handball is a throwing sport, only 14% of acute injuries affected the shoulder. This supports the so-called “shoulder injury paradox” observed in overhead throwing sports. The paradox suggests that adaptive mechanisms and optimized kinetic-chain function may help protect the shoulder from injury despite repetitive loading. A possible explanation may be that beach handball involves fewer high-velocity throws due to limited approach space and reduced throwing stability on sand. Additionally, tactical substitutions and shorter match durations may reduce cumulative throwing load per player. Furthermore, throwing techniques in beach handball (e.g. spin shots, inflight shots) are often performed in mid-air with more trunk involvement and less maximum shoulder acceleration, which may lower peak joint loads and reduce shoulder injury risk. In contrast, shoulder injuries are more common in indoor handball, where higher throwing velocities and more frequent physical contact contribute to increased shoulder stress and injury risk [10, 30, 31].

Among the overuse injuries examined, knee/calf/lower leg injuries accounted for 28% of cases, with particularly high rates of patellar tendinopathy and jumper's knee followed by injuries to the ankle or foot, spine and shoulder. This injury distribution is comparable to the existing literature of indoor handball [17, 30]. Overuse injuries often result from repetitive stress on the knee and lower leg, a common occurrence in sports involving running and jumping. As our results are the first findings on overuse injuries in beach handball and it is well known that overuse injuries can be underreported, further analysis is needed to provide a more precise understanding.

### Injury timing and mechanisms

In the present study, the majority of acute injuries (62%) occurred during competition, with a smaller proportion (38%) reported during training. This competition-related injury rate in beach handball was notably lower compared to indoor handball [2, 19]. This distinction could be attributed to the non-contact rules governing the sport.

Contact with another athlete was the most common injury mechanism with 55% of all acute injuries. Despite the official non-contact rules, incidental and rule-violating contact can still occur and cause injuries, particularly in competitive play. These results are comparable with the existing literature on beach handball [11], but lower than for indoor handball, where injury prevalence rates up to 61% have been reported [3]. Despite the non-contact and fair play rules of beach handball, the results still underscore the importance of physical contact as the main cause of injuries in beach handball. Additionally, approximately 28% of injuries were associated with jumping, whereas 21% of injuries were linked to throwing, passing or blocking the ball. These findings may be due to the physical nature of the sport, where athletes engage in physical contact, particularly following jumps and landings during acrobatic throwing and blocking situations. Strategies to minimize contact-related injuries could include enhanced training in body positioning, adequate distance from opponents during offensive and defensive actions with appropriate timing, safe landing techniques after jumping and enforcing rules. The high incidence of injuries during jump-related actions highlights the importance of proper landing techniques for offense and defense and injury prevention programs targeting lower extremity injuries. The fact that ball-playing mechanisms like throwing, passing and blocking play a role in injuries suggests that skill development and tactical awareness during ball interactions are essential for injury prevention. This might encompass refining shooting techniques, blocking methods, and defensive strategies, consistent with the findings by Hatzimanouil et al. [12].

### Risk factors

The number of months playing beach handball per year was an independent risk factor for any injury as well as overuse injuries specifically. For acute injuries, the number of tournaments per year and older age were independent risk factors. This may be due to factors such as longer exposure to the sport, increased fragility, aged-related degenerative changes in musculoskeletal tissues or changes in playing style with age [21, 25]. Achenbach et al. [2] also reported a higher injury incidence in senior beach handball athletes compared to u-17 athletes. Athletes in the shooting specialist position had a lower risk of overuse injuries in our study. This position may involve less physical contact and fewer high-impact movements such as acrobatic spin or inflight shots, thereby reducing cumulative joint and tissue stress and risk of injury. This contrasts with the findings of Achenbach et al. [2], who reported a high incidence of acute injuries among central defenders and shooting specialists during a single tournament in beach handball, although no time-loss injuries occurred in shooting specialists. It is important to note that Achenbach et al. [2] focused solely on acute injuries within a short time frame, whereas our data were collected retrospectively and include overuse injuries. Differences in study design, injury definitions, contact exposure and the longer observation period in our study may explain the divergent finding. Further studies are needed to better understand positional injury risks. Several factors, including sex, hours played, position, play level and throwing arm did not show a significant association with the risk of injury in the present study and should be investigated further. This lack of effect on the throwing arm despite effected shoulder stress, may be explained by biomechanical adaptions or compensatory mechanisms reducing injury risk [10]. Individual variations and interactions between these factors may have influenced the risk of injury. Therefore, a holistic injury prevention approach that considers athlete-specific factors, physical conditioning, and training regimens is essential in beach handball. Especially, while injury incidence rates did not significantly differ between men and women, it's worth exploring potential gender-specific differences in injury types, mechanisms, or recovery times through further research.

### Return to sport

The vast majority of athletes returned to sport after injury and all remaining athletes only had recent injuries. Most athletes needed two months or less to return to full beach handball training/competition after an injury (acute: 70%; overuse: 80%), with the majority of athletes returning to their previous level of play (self-reported) within the same time frame (acute: 54%, overuse: 60%). This faster RTS in chronic injuries may appear counterintuitive but likely reflects the milder nature of most reported overuse injuries, such as tendinopathies, which often allow continued or only shortly interrupted participation with load management despite mild persistent symptoms. This, combined with the high return to sport rate and level indicated that the majority of injuries were not severe. This study is the first to show the return to sport in beach handball and seems to be lower than the comparable data for indoor handball, with fewer serious injuries and fewer operations [2, 3, 17, 30].

### Limitation

First, this study has limitations regarding the nature of the retrospective data, where all injuries, diagnoses, severity level, and RTS time frames were self-reported without confirmation by a medical professional, potentially limiting the accuracy of the reported injuries and diagnoses. This might have led to an information bias as well as a recall/memory bias and misclassification, particularly for detailed diagnoses and injury mechanisms over a five-year period. However, the self-reporting nature of the questionnaire offered low entry barriers to participate in the study. In prospective follow up studies these potential biases should be reflected.

Second, the questionnaire was only available in English imposing a potential language barrier for non-native-speakers.

Third, the dropout rate during answering of the questionnaire due to the length of the questionnaire may impose a selection bias. However, by designing a detailed questionnaire, the present study was able to gain deeper insights in the reported injuries. The likelihood that athletes without injuries may have been more inclined to complete the shorter questionnaire might have had an influence of the results and should be addressed in future studies.

Fourth, the definition of injury varies significantly among epidemiological studies on beach handball injuries. In our study, we specifically collected data on significant injuries, defined as an absence from training for at least seven days. Although this approach may lead to an underestimation of absolute injury numbers, it allows for a comprehensive understanding of significant injuries and their most frequent mechanisms.

Fifth, most of the included athletes were likely active in both beach and indoor handball, as beach handball is typically played during indoor handball off-season. However, it was not assessed in the questionnaire whether athletes were additionally active in indoor handball. While we explicitly asked for overuse injuries that occurred during the beach handball season, it is possible that some of these were originally caused by indoor handball and carried over into the beach season. This overlap between disciplines must be considered when interpreting overuse injury data.

Sixth, treatment decisions vary between countries and may not directly reflect injury severity or sport-specific demands. However, despite geographic differences the treatment of the injuries shows good results regarding return to sport and patient satisfaction.

Seventh, the use of social media for recruitment may have skewed the sample toward a younger and more digitally engaged population. However, most active beach handball athletes come from an age-group that is active in social media. Additionally, other channels such as coaches, official, and tournament organizers were used to reach a broader athlete population.

Eigth, we considered standardized classification systems like the UEFA model, we ultimately opted for a practical, athlete-reported approach to ensure accessibility and completion by a broad international sample.

## Conclusion

Injury incidence was low among beach handball athletes. Injuries most commonly affected the lower extremity. Older age, number of months playing beach handball and number of tournaments were risk factors for injury. Only 8% of the injuries required surgery, and return to sport outcomes was favorable. This implies a potential for targeted injury prevention strategies and the reassurance of low injury burden in beach handball.

## Supplementary Information

Additional file 1. Questionnaire.

Additional file 2. Diagnoses for acute injuries.

Additional file 3. Diagnoses of overuse injuries.

Additional file 4. Multivariate logistic regression analysis of all injuries.

Additional file 5. Multivariate logistic regression analysis of acute injuries.

Additional file 6. Multivariate logistic regression analysis of overuse injuries.

Additional file 7. Injury timing and location on the field of acute injuries.

Additional file 8. Injury mechanism of acute injuries.

Additional file 9. Treatment after acute injuries.

Additional file 10. Treatment after overuse injuries.

Additional file 11. Return to sport after acute injuries.

Additional file 12. Return to sport after overuse injuries.

## Data Availability

The datasets used and/or analyzed during the current study are available from the corresponding author on reasonable request.

## Abbreviations

RTS: Return to sport
BMI: Body mass indey
RTA: Return to activity
RTC: Return to competition
SD: Standard deviation
OR: Odds ratio
CI: Confidence interval
ACL: Anterior cruciate ligament
