# Supplementary material for: Low injury incidence and excellent return to sport after injuries in beach handball—a cross-sectional survey of 651 athletes
Source: BMC Sports Sci Med Rehabil. 2025 Aug 4;17:224. doi: 10.1186/s13102-025-01252-w (PMC12323119; doi:10.1186/s13102-025-01252-w)
Supplement: Supplementary file 1 — Additional file 1. Questionnaire. [file 13102_2025_1252_MOESM1_ESM.docx]

# Participation

1. Would you like to participate?

- Yes
- No

1. If you are <18 years old, are your parents informed and do they agree to your participation in this survey? (requirement)

- I’m over 18 years old
- Yes, my parent(s) or guardian agree to my anonymous participation
- No

# Demographic data

1. What is your biological gender?

- Male
- Female

1. How old are you?

1

100


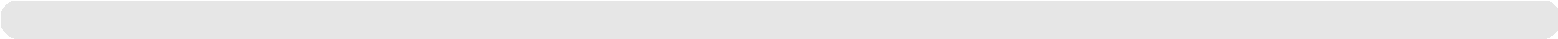

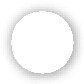

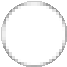


1. In which country do you live?

- Austria
- Angola
- Argentina
- Brazil
- Belarus
- Croatia
- Czech Republic
- Denmark
- Egypt
- France
- Germany
- Hungary
- Iceland
- Japan
- Korea
- Macedonia
- Latvia
- Netherlands
- Norway
- Qatar
- Portugal
- Poland
- Russia
- Slovenia
- Sweden
- Switzerland
- Serbia
- Spain
- Ukraine
- USA

Others

1. How tall are you in cm? (Note: 5“0 = 153 cm; 5“2 = 158 cm; 5“4 = 163 cm; 5“6 = 168 cm 5“8 = 173 cm; 5“10 = 178 cm; 6”0 = 183 cm; 6”2 = 188 cm; 6”4 = 193 cm; 6“6 = 198 cm; 6“8 = 203 cm; 6“10 = 208 cm; 7“0 = 213 cm)

1

250


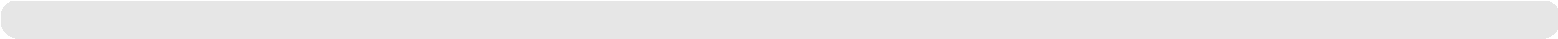

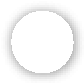

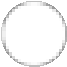


1. How much do you weigh in kg? (Note: One pound = 0.45 Kilograms)

1

180


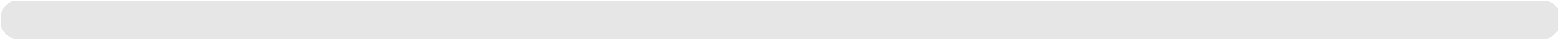

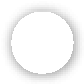

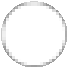


# Sporting activity

1. Do you play Beach Handball?

- Yes
- No

[If questions 8 is answered “no”, end of survey]

# Beachhandball

1. For how many years have you been playing beach handball regularly?

1

100


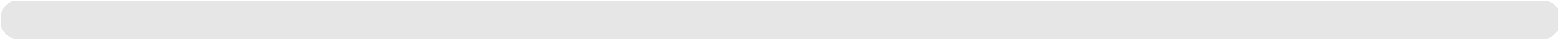

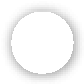

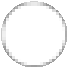


1. How many months a year do you play/train beach handball?

1

100


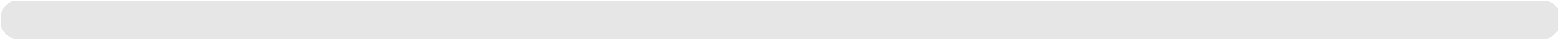

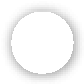

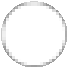


1. How many hours do you play/train beach handball per week when competing?

1

100


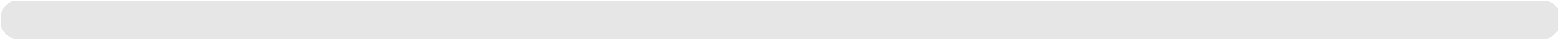

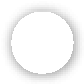

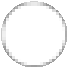


1. How many championships/tournaments do you play per year?

- 0-3
- 3-6
- 6-9
- 10+

1. How many games do you play approximately per year?

- 0-5
- 6-10
- 11-15
- 16-20
- 21-25
- 25+

1. What position do you usually play? (please tick all that apply)

- Goalkeeper
- Defense
- Specialist/Shooter
- Left Wing
- Right wing
- Pivot
- Backfield

1. Which is your throwing arm?

- Left arm
- Right arm

1. At what level do you play?

- I play casually (not in a team)
- I play at a competitive level (in a team)
- I play at a semi-professional level (in a team, receiving money/sponsorship/scholarship)
- I am a professional beach handball player (beach handball is my main occupation)

1. What was the highest level you competed at in the last 5 years?

- Local competitions
- Regional competitions
- Nationwide competitions
- International competitions

# Injuries

1. Have you sustained an injury **while / from playing beach handball** in the **last five years**?

- Yes
- No

1. *(If q18 yes) If you had multiple injuries, please start with the one that affected your life and sports the most; you will get the opportunity to tell us about other injuries later on.*
   What type of injury did you have?

- An acute injury - with a sudden onset of pain/symptoms, associated with one specific identifiable traumatic event (e.g. twisting joint, collision, etc.)
- An overuse injury - with a gradual onset, not associated with one specific identifiable traumatic event responsible for the pain/symptoms

1. *(If q19: acute injury) Did this injury render you unable to play and practice beach handball for a minimum of 7 days?*

- Yes
- No

# Acute Injuries

## Type

1. Please indicate the location of your injury (choose one)

- Head and neck
- Spine (below neck)
- Chest Wall / Torso / Abdomen
- Shoulder
- Elbow / arm
- Hand / wrist
- Hip / pelvis/ thigh
- Knee /calf / lower leg
- Ankle or foot

[Forward to one of questions 26 to 34 depending on the selected location]

1. What kind of head or neck injury did you have?

- *Head:* Broken nose
- *Head:* Broken bone of the face or skull (other than nose)
- *Head: Eye injury*
- *Brain:* Traumatic brain injury with permanent damage
- *Brain:* Concussion
- *Brain:* loss of consciousness (with symptoms possibly lasting for up to 2 weeks)
- *Neck:* Broken vertebrae (bone of the neck)
- *Neck:* Whiplash injury
- *Neck:* Disk injury
- *Neck:* Nerve injury (with loss of sensation (feeling) or muscle power)
- *Soft tissue:* Laceration (wound) of skin
- *Others:* Chipped/broken tooth
- Face injury, not specified
- Eye injury, not specified
- Skull injury, not specified
- Neck injury, not specified

1. What kind of spine injury (below the neck) did you have?

- Broken vertebra (bone)
- Sacral stress fracture
- Disk injury / prolapse
- Muscular back pain
- Contusion / Bruising
- Ligament sprains / whiplash
- Spinal nerve injury with a loss of sensation (feeling) or muscle power
- Laceration/ Skin wound
- Upper back injury, not specified
- Lower back injury, not specified
- Rib injury, not specified
- Spine injury, not specified

1. What kind of torso / chest wall / abdominal injury did you have?

- *Bone:* Rib fracture
- *Bone:* Rib contusion / bruise
- *Joint:* Sternoclavicular joint dislocation/instability (inner collar bone joint injury)
- *Muscle:* Pectoralis major/minor muscle injury
- *Lung:* Pneumothorax (air leaked outside of the lungs)
- *Abdominal:* Injury to a solid organ (that is, liver, spleen, pancreas, or kidneys)
- Abdominal: Injury to a hollow organ (that is, stomach, small intestine, colon, or bladder)
- Abdominal: Injury to the abdominal muscles
- Injury to reproductive organs
- Laceration / Skin wound
- Chest injury, not specified
- Abdominal injury, not specified

1. What kind of shoulder injury did you have?

- *Bone:* Broken humerus (fracture of upper arm at the joint)
- *Bone:* Broken scapula (shoulder blade)
- *Bone:* Broken clavicle (collarbone)
- *Joint:* Dislocated shoulder
- *Joint:* Subjectively „unstable“ shoulder / subluxation
- *Joint:* Separated shoulder (acromioclavicular joint injury/dislocation)
- *Joint:* Sternoclavicular joint dislocation/instability (inner collar bone joint injury)
- *Joint:* Bursitis / tendinitis of the rotator cuff
- *Muscle:* Pectoralis major/minor muscle injury
- *Tendon:* Rotator cuff tear
- *Tendon:* Impingement Syndrome
- *Tendon:* SLAP-tear (tear of the upper glenoid labrum - rim around the socket - where the long head of biceps tendon attaches)
- *Tendon:* Long head of the Biceps tendinitis
- *Functional:* Scapular dyskinesia / malposition / “SICK shoulder” (movement disorder or asymmetry of shoulder bone)
- *Nerve:* Suprascapular neuropathy / palsy (atrophy of infraspinatus muscle with decreased external rotation force
- *Soft tissue:* Muscle strain/tear
- *Soft tissue:* Laceration (wound) of skin
- *Soft* tissue: Contusion / bruise
- Shoulder injury, not specified

1. What kind of elbow or arm injury did you have?

- *Bone:* Broken ulna (forearm)
- *Bone:* Broken radius (forearm)
- *Bone:* Broken humerus (upper arm, excluding shoulder region)
- *Joint:* Elbow dislocation
- *Ligament:* Radial Collateral Ligament (RCL) injury
- *Ligament:* Thrower's elbow (sprain injury of the ulnar collateral ligament)
- *Tendon:* Epicondylitis / tennis or golfer‘s elbow
- *Tendon:* Biceps tendon rupture (at the elbow)
- *Tendon:* Biceps tendonitis (at the elbow)
- *Tendon:* Triceps tendon rupture
- *Tendon:* Triceps tendonitis
- *Nerve:* Ulnar nerve inflammation
- *Soft tissue:* Muscle strain/tear
- *Soft tissue:* Laceration / Skin wound
- *Soft tissue:* Contusion / bruise
- Elbow injury, not specified
- Upper arm injury, not specified
- Forearm injury, not specified

1. What kind of acute hand or wrist injury did you have?

- *Bone:* Broken ulna (forearm bone at the wrist)
- *Bone:* Broken radius (forearm bone at the wrist)
- *Bone:* Broken carpal bone at the wrist (small bones of the hand)
- *Bone:* Broken metacarpal bone (long bones in the palm of the hand)
- *Bone:* Broken finger
- *Joint:* Wrist sprain
- *Joint:* Finger dislocation
- *Joint:* Jammed finger
- *Base of the thumb:* Gamekeeper's thumb / skier's thumb / UCL tear (injury to the ulnar collateral ligament (UCL)
- *Tendon:* Mallet finger / hammer finger (inability to straighten the fingertip due to an extensor tendon injury)
- *Tendon:* Jersey finger / rugby finger (Inability to flex the fingertip due to a flexor tendon injury)
- *Nerve:* Palm nerve injury (De Quervain Tenosynovitis)
- *Soft tissue: Laceration / Skin wound*
- *Wrist / metacarpal injury, not specified*
- *Finger injury, not specified*

1. What kind of hip / pelvic / thigh injury did you have?

- *Bone:* Broken femur (thigh bone)
- *Bone:* Broken pelvic bone
- *Bone:* Tailbone injury
- *Joint:* Labral tear (cartilage rim around hip socket)
- *Joint:* Femoroacetabular impingement (deformity of the neck of the femur)
- *Muscle:* Pulled/ Torn/ Strained hamstring muscle
- *Muscle:* Avulsion / tear off from the bone of the quadriceps muscle at the hip
- *Soft tissue:* Runners hip (trochanteric bursitis at the outside of the hip)
- *Soft tissue:* Snapping hip syndrome / dancer's hip (inflammation and snapping sensation when bending and stretching the hip)
- *Soft tissue:* Piriformis syndrome (compression of the sciatic nerve by the piriformis muscle)
- Pelvis injury, not specified
- Hip injury, not specified
- Thigh injury, not specified

1. What kind of knee or lower leg injury did you have?

- *Bone:* Broken femur (thigh)
- *Bone:* Broken tibia (lower leg)
- *Bone:* Broken fibula (lower leg)
- *Ligament:* Anterior cruciate ligament (ACL) **tear**
- *Ligament:* Posterior cruciate ligament (PCL) **tear**
- *Ligament:* Medial/inner collateral ligament (MCL) **tear**
- *Ligament:* Lateral/outer collateral ligament (LCL) **tear**
- *Ligament:* Anterior cruciate ligament (ACL) **sprain**
- *Ligament:* Posterior cruciate ligament (PCL) **sprain**
- *Ligament:* Medial/inner collateral ligament (MCL) **sprain**
- *Ligament:* Lateral/outer collateral ligament (LCL) **sprain**
- *Ligament:* Runner’s knee / Iliotibial ligament syndrome (inflammation on the outside of the knee due to friction between the iliotibial ligament and the outside femur)
- *Joint:* Torn meniscus
- *Joint:* Cartilage injury
- *Joint:* Kneecap dislocation
- *Tendon:* Patellar tendinitis/ jumper’s knee
- *Tendon:* Pes anserinus syndrome (inflammation of the tendon attachments at the lower inner side of the knee)
- *Muscle:* Quadriceps tendon (thigh muscle) tear at the knee
- *Muscle:* Calf muscle injury
- Knee injury, not specified
- Lower leg injury, not specified

1. What kind of ankle / foot injury did you have?

- *Bone:* Broken tibia (lower leg bone at the ankle)
- *Bone:* Broken fibula (lower leg bone at the ankle)
- *Bone:* Broken bone of the foot
- *Bone:* Broken toe
- *Joint:* Cartilage injury
- *Ligaments:* Ankle **sprain** lateral / outside
- *Ligaments:* Ankle **sprain** medial / inside
- *Ligaments:* Outer/ Lateral ankle ligament **tear** or bony avulsion
- *Ligaments:* Inner/ Medial ankle ligament **tear** or bony avulsion
- *Tendon:* Achilles tendinitis (inflammation)
- *Tendon:* Achilles tendon rupture
- *Soft tissue:* Sand toe (hyperflexion/ downward-flexion of the toe, resulting in an injury of the dorsal/upper joint capsule)
- Ankle injury, not specified
- Foot injury, not specified
- Toe injury, not specified

## Risk factors

1. What mechanism caused your injury? (please tick all that apply)

- *Jump:* Coming down on the sand after a jump
- *Jump:* Coming down on a team mate after a jump
- *Jump:* Coming down on an opponent after a jump
- *Contact:* Hit by a team mate coming down after a jump
- *Contact:* Hit by an opponent coming down after a jump
- *Contact:* Collision with a team mate
- *Contact:* Collision with an opponent (offense)
- *Contact:* Collision with an opponent (defense)
- *Contact:* Contact with the ball
- *Contact:* Contact with the sand
- *Contact:* Contact with the boundary line
- *Contact:* Contact with out-of-bounds apparatus
- *Playing the ball:* Whilst blocking
- *Playing the ball:* Whilst passing
- *Playing the ball:* Whilst throwing 1 point shot, specialist shot
- *Playing the ball:* Whilst throwing a 360°
- *Playing the ball:* Whilst throwing an inflight
- *Others:* Unintentionally hit by ball
- *Others:* Falling
- *Others:* Rapid change of direction
- *Others:* Rotation around planted foot
- *Others:* Acute overwork
- Others: (please specify)

1. Where on the court did your injury occur?

- Attacking third
- Middle third
- Defensive third
- Out of bounds

1. Did this injury occur during training or competition?

- Training
- Competition

1. When did the injury happen?

- During warm-up
- During the first 10 minutes game
- During the last 10 minutes game
- During the first 15 minutes of training
- During the (preplanned) last 15 minutes of training
- In between the first and last 15 minutes of training (middle)
- During shoot-out
- During cool down
- I don’t remember

# Overuse Injury

## Type

1. Please indicate the location of your injury (choose one)

- Head and neck
- Spine (below neck)
- Chest Wall / Torso / Abdomen
- Shoulder
- Elbow / arm
- Hand / wrist
- Hip / pelvis/ thigh
- Knee /calf / lower leg
- Ankle or foot

[Forward to one of questions 44 to 51 depending on the selected location]

1. What kind of neck injury did you have?

- (Chronic) disk injury
- (Chronic) nerve injury
- (Chronic) muscular neck pain
- (Chronic) ligament sprains
- Neck injury, not specified

1. What kind of back / spine injury (lumbar or thoracic) did you have?

- (Chronic) spondylolisthesis (instability between vertebrae, possibly caused by hyperextension)
- Vertebral stress fracture
- Sacral stress fracture
- Scoliosis (S-shaped spine deformity)
- (Chronic) disk injury / prolapse (slipped disc)
- (Chronic) muscular back pain
- (Chronic) nerve injury
- Rib injury, not specified
- Spine injury, not specified

1. What kind of shoulder injury did you have?

- *Joint:* Recurrent shoulder dislocation
- *Joint:* Recurrent feeling of shoulder instability / subluxation
- *Joint:* Chronic acromioclavicular joint (at outer end of collar bone) injury/dislocation/instability (“shoulder separation”)
- *Joint:* Recurrent sternoclavicular joint (at inner end of collar bone) dislocation/subluxation/instability
- *Joint*: Bursitis
- *Joint:* Shoulder impingement syndrome (compression of bursa and rotator cuff tendons)
- *Joint:* Glenohumeral internal rotation deficit (GIRD) / (rear) shoulder capsule contraction
- *Joint:* SLAP-tear (tear of the upper glenoid labrum - rim around the socket - where the long head of biceps tendon attaches)
- *Tendon:* long head of the biceps tendonitis
- *Tendon*: (Partial) rotator cuff tear (causing pain / weakness)
- *Nerve*: (Chronic) suprascapular nerve injury /neuropathy / palsy (atrophy of infraspinatus shoulder muscle with decreased external rotation force)
- *Functional:* Shoulder blade malposition / scapular dyskinesia /SICK shoulder
- *Soft tissue:* Chronic muscle strain/tear
- Other, not specified

1. What kind of elbow or arm injury did you have?

- *Tendon:* Tennis elbow - radial (outer) epicondylitis
- *Tendon:* Golfer‘s elbow - ulnar (inner) epicondylitis
- *Tendon:* Biceps tendonitis (at the front of the elbow)
- *Tendon:* Triceps tendon inflammation / tendonitis
- *Ligaments:* Recurrent subjective (feeling of) elbow instability caused by thrower's elbow / ulnar collateral ligament (UCL) injury
- *Ligaments:* Recurrent subjective (feeling of) elbow instability caused by radial collateral ligament (RCL) injury
- *Ligaments:* Recurrent subjective (feeling of) elbow instability (cannot specify the type)
- *Joint:* Cartilage damage
- *Nerve:* Ulnar nerve inflammation
- *Soft tissue:* Recurrent muscle strain/tear
- Elbow injury, not specified
- Upper arm injury, not specified
- Forearm injury, not specified

1. What kind of hand or wrist injury did you have?

- *Bone*: Stress fracture
- *Joint:* Recurrent wrist pain
- *Joint:* Recurrent finger dislocation
- *Ligaments:* Recurrent gamekeeper's thumb / skier's thumb / UCL tear (injury to the ulnar collateral ligament (UCL) of the base of the thumb)
- *Tendon:* Recurrent mallet finger / hammer finger (inability to straighten the fingertip due to an extensor tendon injury)
- *Tendon:* Recurrent jersey finger / rugby finger (Inability to flex the fingertip due to a flexor tendon injury)
- *Nerve:* De Quervain Tenosynovitis (palm nerve injury)
- *Wrist / metacarpal injury, not specified*
- *Finger injury, not specified*

1. What kind of pelvis/hip or thigh injury did you have?

- *Bone:* Tailbone injury
- *Joint:* Femoroacetabular impingement (deformity of the neck of the femur)
- *Joint:* labral tear (cartilage rim around hip socket)
- *Muscle:*  Hamstring muscle strain/partial tear
- *Muscle:* Other muscle strain
- *Soft tissue:* Bursitis
- *Soft tissue:* Snapping hip syndrome / dancer's hip (snapping sensation when bending and stretching the hip)
- *Soft tissue:* Piriformis syndrome (compression of the sciatic nerve by the piriformis muscle)
- Pelvis injury, not specified
- Hip injury, not specified
- Thigh injury, not specified

1. What kind of knee or lower leg injury did you have?

- *Joint:* Instability to the lateral side (joint twisting to the outside)
- *Joint:* Instability to the medial side (joint twisting to the inside)
- *Joint:* Meniscus tear
- *Joint:* Untreated cartilage injury
- *Joint:* Recurrent kneecap dislocation / patella instability
- *Tendon:* Patella tendinitis/ jumper’s knee
- *Tendon:* Pes anserinus syndrome (inflammation of the tendon attachments at the lower inner side of the knee)
- *Tendon:* Runner’s knee / iliotibial ligament syndrome (inflammation on the outside of the knee due to friction between the iliotibial ligament and the outside femur)
- *Tendon:* Synovitis (inflammation of the inside of the joint capsule)
- Shin splints (pain along the edge of the shinbone)
- *Muscle:* (Chronic) lower leg muscle sprain / inflammation (e.g. calf)
- Knee injury, not specified
- Lower leg injury, not specified
- Upper leg injury, not specified

1. What kind of ankle / foot injury did you have?

- *Bone:* Stress fracture due to chronic overload (not one specific impact)
- *Ligaments:* Recurrent ankle instability leading to sprains/twists to the outside (lateral)
- *Ligaments:* Recurrent ankle instability leading to sprains/twists to the inside (medial)
- *Ligaments:* Recurrent sand toe (hyperflexion of the toe, resulting in pain at the dorsal/upper capsule)
- *Joint:* Cartilage injury
- *Tendon:* Achilles tendinitis
- *Tendon:* Chronic partial Achilles tendon rupture
- Plantar fasciitis
- Heel spur
- Ankle injury, not specified
- Foot injury, not specified
- Toe injury, not specified

## Treatment after an injury

1. (only for acute injuries) How was your injury treated immediatly? (please tick all that apply)

- I carried on playing
- Ice was applied
- compression was applied
- the limb was elevated
- a splint was applied to immobilize the injured area
- I went/was taken to hospital
- Nothing, I just stopped playing

1. How was your injury treated? (please tick all that apply)

- Rest / Waited it out (missed training and competitions)
- Regular painkillers
- Anti-inflammatory medication
- Injections/ infiltrations (e.g. into torn muscle or into inflamed area)
- Physiotherapy
- Stabilization in a brace
- Immobilization in a Splint
- Immobilization in a Cast
- Surgery
- Other (please specify)

1. What was the treatment response to your injury? (please tick all that apply)

- *Reduce stress:* Rest; break from training / games / competition
- *Reduce stress:* Reduction in training **intensity**
- *Reduce stress:* Reduction in game / competition **intensity**
- *Reduce stress:* Reduction in training **frequency**
- *Reduce stress:* Reduction in game / competition **frequency**
- *Reduce stress:* Reduction of intensity of training exercises especially affecting injured site
- *Reduce stress:* Reduction of physical activity in the **workplace**
- *Regeneration:* Increased / more frequent **breaks** between training sessions
- *Regeneration:* Increase in sleep
- *Regeneration:* **Dietary** change or supplementation
- *Modifications:* Modification of training exercises
- *Modifications:* Change in position
- *Prevention:* Increase of **stretching** exercises
- *Prevention:* Increase of **strength training** exercises
- *Prevention:* Increase of **proprioception**/neuromuscular training exercises
- *Medical:* Physiotherapy
- *Medical:* **Injections**/ infiltrations (e.g. into torn muscle or into inflamed area)
- *Medical:* Surgery
- *Gear:* Wearing a brace
- *Gear:* Taping
- I stopped playing permanently
- I carried on playing as usual and the injury resolved by itself
- Nothing applies

# Descriptive data on the injury

1. Did this injury occur to your dominant or non-dominant side?

- Dominant side
- Non-dominant side
- Doesn't apply

1. Is this a recurring injury?

- I have had the same injury before.
- I have had the same injury again since the time I am referring to
- No, this is the first and only time I have been injured in this way at this site.

1. How long ago was the onset of this injury?

- Less than a month
- 1 – 3 months
- 3 – 6 months
- 6 – 12 months
- ~ 1,5 years
- ~ 2 years
- ~ 2,5 years
- ~ 3 years
- ~ 3,5 years
- ~ 4 years
- ~ 4,5 years
- ~ 5 years

# Return to sports

1. Have you returned to playing beach handball since your injury?

- Yes
- No

1. *(if q50: no)* What has kept you from going back?

- My injury was recent, I’m still expecting to return
- Pain
- Loss of range of motion (of a joint)
- Persisting instability
- Fear of getting injured again
- Loss of position on team
- Unable to regain previous form
- Doctor’s advice

1. How long did it take to return to light training/ activity? (e.g. jogging, light training)

- My injury did not make me stop light training / activity at any point
- Less than 1 week
- 1 – 4 weeks
- ~ 2 months
- ~ 3 months
- ~ 4 months
- ~ 5 months
- ~ 6 months
- ~ 7 months
- ~ 8 months
- ~ 9 months
- ~ 10 months
- ~ 11 months
- ~ 12 months
- > 1 year
- I did not return yet, but I think I will
- I did not return and I don’t think I will

1. How long did it take to return to full beach handball training / competition?

- My injury did not make me stop beach handball training / competition at any point
- Less than 1 week
- 1 – 4 weeks
- ~ 2 months
- ~ 3 months
- ~ 4 months
- ~ 5 months
- ~ 6 months
- ~ 7 months
- ~ 8 months
- ~ 9 months
- ~ 10 months
- ~ 11 months
- ~ 12 months
- > 1 year
- I did not return yet, but I think I will
- I did not return and I don’t think I will

1. How long did it take you to return to the physical level you were at before your injury?

- My injury did not make me perform beach handball at a lower level at any point
- Less than 1 week
- 1 – 4 weeks
- ~ 2 months
- ~ 3 months
- ~ 4 months
- ~ 5 months
- ~ 6 months
- ~ 7 months
- ~ 8 months
- ~ 9 months
- ~ 10 months
- ~ 11 months
- ~ 12 months
- More than a year
- I am still not at my previous level
- I did not return yet, but I think I will
- I did not return and I don’t think I will

# Further injury

1. Did you have another injury whilst playing beach handball in the last five years?

- Yes.
- No.

1. *(If q55 yes) Please continue with one that affected your life and sports the second-most; you will get the opportunity to tell us about other injuries later on.*
   What type of injury did you have?

- An acute injury - with a sudden onset of pain/symptoms, associated with one specific identifiable traumatic event (e.g. wrong movement, collision, etc.)
- An overuse injury - with a gradual onset, not associated with one specific identifiable traumatic event responsible for the pain/symptoms

*[Repeat questions 20 through 56 until the answer to question 56 is “no”.]*
