# Supplementary material for: Low injury incidence and excellent return to sport after injuries in beach handball—a cross-sectional survey of 651 athletes
Source: BMC Sports Sci Med Rehabil. 2025 Aug 4;17:224. doi: 10.1186/s13102-025-01252-w (PMC12323119; doi:10.1186/s13102-025-01252-w)
Supplement: Supplementary file 2 — Additional file 2. Diagnoses for acute injuries. [file 13102_2025_1252_MOESM2_ESM.docx]

| **Injury Location** | **Total number (n=102)** | **Percentage** |
| --- | --- | --- |
| **Ankle or foot** | **32** | **31.4** |
| Bone: Broken toe | 9 | 8.8 |
| Ligaments: Outer/ Lateral ankle ligament tear or bony avulsion | 6 | 5.9 |
| Ankle injury, unspecified | 5 | 4.9 |
| Ligaments: Ankle sprain lateral / outside | 4 | 3.9 |
| Foot injury, unspecified | 3 | 2.9 |
| Toe injury, unspecified | 2 | 2.0 |
| Bone: Broken bone of the foot | 1 | 1.0 |
| Lost Toenail | 1 | 1.0 |
| Soft tissue: Sand toe (hyperflexion/ downward-flexion of the toe, resulting in an injury of the dorsal/upper joint capsule) | 1 | 1 |
| **Knee / calf / lower leg** | **25** | **24.5** |
| Muscle: Calf muscle injury | 5 | 4.9 |
| Muscle: Quadriceps tendon (thigh muscle) tear at the knee | 3 | 2.9 |
| Meniscal tear | 2 | 2.0 |
| Knee injury, unspecified | 2 | 2.0 |
| Bone: Broken tibia (lower leg) | 1 | 1.0 |
| Haematoma | 1 | 1.0 |
| Hamstring tear, bruised thigh | 1 | 1.0 |
| Joint: Cartilage injury | 2 | 2.0 |
| Knee to knee contact causing bursitis | 1 | 1.0 |
| Ligament: Anterior cruciate ligament (ACL) sprain | 1 | 1.0 |
| Ligament: Medial/inner collateral ligament (MCL) tear | 1 | 1.0 |
| Ligament: Posterior cruciate ligament (PCL) tear | 1 | 1.0 |
| Lower leg injury, unspecified | 1 | 1.0 |
| Upper leg injury, unspecified | 1 | 1.0 |
| Shinbone/lower knee injury by collision | 1 | 1.0 |
| Meniscus bruise | 1 | 1.0 |
| **Shoulder** | **14** | **13.7** |
| Joint: Dislocated shoulder | 5 | 4.9 |
| Tendon: SLAP-tear (tear of the upper glenoid labrum - rim around the socket - where the long head of biceps tendon attaches) | 4 | 3.9 |
| Tendon: Rotator cuff tear | 2 | 2.0 |
| Bone: Broken clavicle (collarbone) | 1 | 1.0 |
| Joint: Separated shoulder (acromioclavicular joint injury/dislocation) | 1 | 1.0 |
| Tendon, GLAD tear ; partial dislocation of the shoulder | 1 | 1.0 |
| **Hand / wrist** | **11** | **10.8** |
| Bone: Broken metacarpal bone (long bones in the palm of the hand) | 3 | 2.9 |
| Joint: Finger dislocation | 2 | 2.0 |
| Base of the thumb: Gamekeeper's thumb / skier's thumb / UCL tear (injury to the ulnar collateral ligament (UCL) | 1 | 1.0 |
| Bone: Broken finger | 1 | 1.0 |
| Bone: Broken radius (forearm bone at the wrist) | 1 | 1.0 |
| Joint: Jammed finger | 1 | 1.0 |
| Tendon: Mallet finger / hammer finger (inability to straighten the fingertip due to an extensor tendon injury) | 1 | 1.0 |
| Finger injury, unspecified | 1 | 1.0 |
| **Head / neck** | **10** | **9.8** |
| Brain: Concussion | 3 | 2.9 |
| Neck: Nerve injury (with loss of sensation (feeling) or muscle power) | 2 | 2.0 |
| Broke two teeth | 1 | 1.0 |
| Head: Broken bone of the face or skull (other than nose) | 1 | 1.0 |
| Head: Broken nose | 1 | 1.0 |
| Nose injury, cut | 1 | 1.0 |
| Face injury, unspecified | 1 | 1.0 |
| **Hip / pelvis / thigh** | **4** | **3.9** |
| Muscle: Pulled/ Torn/ Strained hamstring muscle | 3 | 2.9 |
| Joint: Labral tear (cartilage rim around hip socket) | 1 | 1.0 |
| **Elbow / arm** | 3 | 2.9 |
| Joint: Elbow dislocation | 2 | 2.0 |
| Ligament: Radial Collateral Ligament (RCL) injury | 1 | 1.0 |
| **Spine (below neck)** | 2 | 2.0 |
| Compression of lower spine | 1 | 1.0 |
| Muscular back pain | 1 | 1.0 |
| **Chest wall / torso / abdomen** | 1 | 1.0 |
| Muscle: Pectoralis major/minor muscle injury | 1 | 1.0 |

Categorical variables are shown as number and corresponding percentages.
