# Supplementary material for: Low injury incidence and excellent return to sport after injuries in beach handball—a cross-sectional survey of 651 athletes
Source: BMC Sports Sci Med Rehabil. 2025 Aug 4;17:224. doi: 10.1186/s13102-025-01252-w (PMC12323119; doi:10.1186/s13102-025-01252-w)
Supplement: Supplementary file 3 — Additional file 3. Diagnoses of overuse injuries. [file 13102_2025_1252_MOESM3_ESM.docx]

| **Injury location** | **Total number (n=72)** | **Percentage** |
| --- | --- | --- |
| **Knee / calf/ lower leg** | **20** | **27.8** |
| Tendon: Patellar tendinopathy/ jumper’s knee | 8 | 11.1 |
| Knee injury, unspecified | 4 | 5.6 |
| Joint: Meniscus tear | 3 | 4.2 |
| Lower leg injury, unspecified | 2 | 2.8 |
| Knee pain, unspecified | 1 | 1.4 |
| Tendon: Runner’s knee/iliotibial ligament syndrome (inflammation on the outside of the knee due to friction between the iliotibial ligament and the outside femur) | 1 | 1.4 |
| Open bruise and open wounds covered in gravel from repeated scraping | 1 | 1.4 |
| **Ankle or foot** | **15** | **20.8** |
| Bone: Stress fracture due to chronic overload (not one specific impact) | 4 | 5.6 |
| Tendon: Achilles tendinopathy | 4 | 5.6 |
| Toe injury, unspecified | 2 | 2.8 |
| Foot injury, unspecified | 1 | 1.4 |
| Inflammation of the peroneal tendon | 1 | 1.4 |
| Ligaments: Recurrent ankle instability leading to sprains/twists to the inside (medial) | 1 | 1.4 |
| Ligaments: Recurrent ankle instability leading to sprains/twists to the outside (lateral) | 1 | 1.4 |
| Plantar fasciitis | 1 | 1.4 |
| **Spine (below neck)** | **11** | **15.3** |
| (Chronic) muscular back pain | 4 | 5.6 |
| (Chronic) disk injury / prolapse (slipped disc) | 2 | 2.8 |
| Scoliosis (S-shaped spine deformity) | 2 | 2.8 |
| (Chronic) spondylolisthesis (instability between vertebrae, possibly caused by hyperextension) | 2 | 2.8 |
| Sacral stress fracture | 1 | 1.4 |
| **Shoulder** | **9** | **12.5** |
| Joint: Bursitis | 4 | 5.6 |
| Tendon: (Partial) rotator cuff tear (causing pain / weakness) | 2 | 2.8 |
| Soft tissue: Chronic muscle strain/tear | 2 | 2.8 |
| Joint: SLAP-tear (tear of the upper glenoid labrum - rim around the socket - where the long head of biceps tendon attaches) | 1 | 1.4 |
| **Hip / pelvis / thigh** | **6** | **8.3** |
| Muscle: Hamstring muscle strain/partial tear | 2 | 2.8 |
| Muscle: Other muscle strain | 2 | 2.8 |
| Joint: labral tear (cartilage rim around hip socket) | 1 | 1.4 |
| Pelvis injury, unspecified | 1 | 1.4 |
| **Elbow/arm** | **5** | **6.9** |
| Tendon: Golfer’s elbow - ulnar (inner) epicondylitis | 2 | 2.8 |
| Tendon: Biceps tendonitis (at the front of the elbow) | 1 | 1.4 |
| Tendon: Tennis elbow – radial (outer) epicondylitis | 1 | 1.4 |
| Tendon: Triceps tendon inflammation / tendonitis | 1 | 1.4 |
| **Hand / wrist** | **4** | **5.6** |
| Finger injury, unspecified | 2 | 2.8 |
| Joint: Recurrent wrist pain | 1 | 1.4 |
| Ligaments: Recurrent gamekeeper's thumb / skier's thumb / UCL tear (injury to the ulnar collateral ligament (UCL) of the base of the thumb) | 1 | 1.4 |
| **Head / neck** | **2** | **2.8** |
| (Chronic) nerve injury | 1 | 1.4 |
| Broken nose | 1 | 1.4 |

Categorical variables are shown as number and corresponding percentages.
