# Supplementary material for: Low injury incidence and excellent return to sport after injuries in beach handball—a cross-sectional survey of 651 athletes
Source: BMC Sports Sci Med Rehabil. 2025 Aug 4;17:224. doi: 10.1186/s13102-025-01252-w (PMC12323119; doi:10.1186/s13102-025-01252-w)
Supplement: Supplementary file 4 — Additional file 4. Multivariate logistic regression analysis of all injuries. [file 13102_2025_1252_MOESM4_ESM.docx]

| **Variable** | **Odds Ratio** | **95% Confidence Interval** | | **p-value** |
| --- | --- | --- | --- | --- |
| Sex (1 = male, 2 = female) | 1.199 | .811 | 1.772 | .362 |
| Age | 1.026 | .997 | 1.056 | .077 |
| BMI | .987 | .920 | 1.059 | .713 |
| Years played beach handball | .992 | .951 | 1.034 | .701 |
| Months playing beach handball per year | 1.113 | 1.039 | 1.192 | **.002** |
| Throwing arm (1 = right, 2 = left) | 1.287 | .445 | 2.102 | .932 |
| Play hours per week (1 = 0-3, 2 = 3-6 , 3 = 6-9, 4 = 10+) | 1.085 | .872 | 1.350 | .464 |
| Tournaments per year (1 = 0-3, 2 = 3-6, 3 = 6-9, 4 = 10+) | 1.111 | .327 | 3.775 | .866 |
| Games per year (1=0-5, 2 = 6-10, 3 = 11-15, 4 = 16-20, 5 = 21-25, 6 = 25+) | 1.490 | .814 | 1.114 | .538 |
| Play level (1=amateur, 2=competitive, 3= semi-professional, 4=professional) | .967 | .858 | 1.797 | .250 |
| Competition level (1 = local, 2 = regional, 3 = national, 4 = international) | 1.242 | .919 | 1.592 | .175 |
| Position |  |  |  |  |
| Goalkeeper | .952 | .758 | 2.533 | .289 |
| Defense | 1.386 | .936 | 2.129 | .100 |
| Shooting specialist | 1.411 | .419 | 1.043 | .075 |
| Backfield | .661 | .561 | 2.136 | .792 |
| Left wing | 1.094 | .621 | 1.542 | .925 |
| Right wing | .978 | .654 | 1.851 | .719 |
| Pivot | 1.100 | .810 | 2.043 | .285 |

Bolded p-values indicate statistical significance.
