# Supplementary material for: Low injury incidence and excellent return to sport after injuries in beach handball—a cross-sectional survey of 651 athletes
Source: BMC Sports Sci Med Rehabil. 2025 Aug 4;17:224. doi: 10.1186/s13102-025-01252-w (PMC12323119; doi:10.1186/s13102-025-01252-w)
Supplement: Supplementary file 5 — Additional file 5. Multivariate logistic regression analysis of acute injuries. [file 13102_2025_1252_MOESM5_ESM.docx]

| **Variable** | **Odds Ratio** | **95% Confidence Interval** | | **p-value** |
| --- | --- | --- | --- | --- |
| Sex (1=male, 2=female) | 1.067 | .657 | 1.733 | .795 |
| Age | 1.048 | 1.012 | 1.085 | **.008** |
| BMI | .927 | .844 | 1.018 | .114 |
| Years played beach handball | .976 | .927 | 1.028 | .360 |
| Months playing beach handball per year | 1.083 | .997 | 1.177 | .059 |
| Throwing arm (1 = right, 2 = left) | .953 | .373 | 2.439 | .921 |
| Play hours per week (1=0-3, 2=3-6, 3=6-9, 4=10+) | 1.007 | .774 | 1.311 | .957 |
| Tournaments per year (1 = 0-3, 2 = 3-6, 3 = 6-9, 4 = 10+) | 1.529 | 1.087 | 2.150 | **.015** |
| Games per year (1=0-5, 2 = 6-10, 3 = 11-15, 4 = 16-20, 5 = 21-25, 6 = 25+) | .994 | .828 | 1.193 | .947 |
| Play level (1=amateur, 2=competitive, 3= semi-professional, 4=professional) | 1.136 | .728 | 1.774 | .575 |
| Competition level (1 = local, 2 = regional, 3 = national, 4 = international) | 1.326 | .936 | 1.879 | .112 |
| Position |  |  |  |  |
| Goalkeeper | 1.088 | .500 | 2.370 | .832 |
| Defense | 1.447 | .872 | 2.402 | .152 |
| Shooting specialist | .835 | .482 | 1.448 | .521 |
| Backfield | 1.065 | .471 | 2.404 | .880 |
| Left wing | .809 | .461 | 1.420 | .460 |
| Right wing | 1.012 | .527 | 1.943 | .971 |
| Pivot | 1.347 | .761 | 2.384 | .307 |

Bolded p-values indicate statistical significance.
