# Supplementary material for: Low injury incidence and excellent return to sport after injuries in beach handball—a cross-sectional survey of 651 athletes
Source: BMC Sports Sci Med Rehabil. 2025 Aug 4;17:224. doi: 10.1186/s13102-025-01252-w (PMC12323119; doi:10.1186/s13102-025-01252-w)
Supplement: Supplementary file 6 — Additional file 6. Multivariate logistic regression analysis of overuse injuries. [file 13102_2025_1252_MOESM6_ESM.docx]

| **Variable** | **Odds Ratio** | **95% Confidence Interval** | | **p-value** |
| --- | --- | --- | --- | --- |
| Sex (1=male, 2=female) | 1.258 | .725 | 2.182 | .415 |
| Age | 1.000 | .958 | 1.044 | .996 |
| BMI | 1.044 | .951 | 1.146 | .365 |
| Years played beach handball | 1.009 | .952 | 1.069 | .769 |
| Months playing beach handball per year | 1.135 | 1.038 | 1.242 | **.005** |
| Throwing arm (right=1, left=2) | 1.129 | .376 | 3.390 | .829 |
| Play hours per week (1=0-3, 2=3-6, 3=6-9, 4=10+) | 1.229 | .913 | 1.655 | .175 |
| Tournaments per year (1 = 0-3, 2 = 3-6, 3 = 6-9, 4 = 10+) | 1.037 | .686 | 1.567 | .863 |
| Games per year (1=0-5, 2 = 6-10, 3 = 11-15, 4 = 16-20, 5 = 21-25, 6 = 25+) | .933 | .750 | 1.160 | .531 |
| Play level (1=amateur, 2= competitive, 3= semi-professional, 4=professional) | 1.278 | .768 | 2.125 | .345 |
| Competition level (1=local, 2=regional, 3=nationwide, 4 = international) | 1.146 | .782 | 1.680 | .484 |
| Position |  |  |  |  |
| Goalkeeper | 1.944 | .868 | 4.355 | .016 |
| Defense | 1.408 | .786 | 2.521 | .250 |
| Shooting specialist | .453 | .225 | .912 | **.027** |
| Backfield | 1.230 | .489 | 3.094 | .660 |
| Left wing | 1.453 | .768 | 2.749 | .251 |
| Right wing | 1.188 | .590 | 2.390 | .630 |
| Pivot | 1.295 | .688 | 2.437 | .423 |

Bolded p-values indicate statistical significance.
