# Supplementary material for: Low injury incidence and excellent return to sport after injuries in beach handball—a cross-sectional survey of 651 athletes
Source: BMC Sports Sci Med Rehabil. 2025 Aug 4;17:224. doi: 10.1186/s13102-025-01252-w (PMC12323119; doi:10.1186/s13102-025-01252-w)
Supplement: Supplementary file 7 — Additional file 7. Injury timing and location on the field of acute injuries. [file 13102_2025_1252_MOESM7_ESM.docx]

| **Injury description** |  | **Total number (n=102)** | **Percentage** |
| --- | --- | --- | --- |
| **Training related injuries** |  | 36 | 35.3 |
|  | During the first 15 minutes of training | 4 | 3.9 |
|  | In between the first and last 15 minutes of training (middle) | 14 | 13.7 |
|  | During the (preplanned) last 15 minutes of training | 9 | 8.8 |
| **Competition related injuries** |  | 66 | 64.7 |
|  | During warm-up | 4 | 3.9 |
|  | During the first 10 minutes of the game | 30 | 29.4 |
|  | During the last 10 minutes of the game | 28 | 27.5 |
|  | During shoot-out | 2 | 2.0 |
|  | I don‘t remember | 11 | 10.8 |
| **Injury location on the field** |  |  |  |
|  | In defense | 44 | 43.1 |
|  | In offense | 44 | 43.1 |
|  | Midcourt | 7 | 6.9 |
|  | Out of bounds | 2 | 2.0 |
|  | I don't remember | 5 | 4.9 |
| **Side of extremity** |  |  |  |
|  | Dominant side | 45 | 44.1 |
|  | Non-dominant side | 33 | 32.4 |
|  | Doesn't apply | 24 | 23.5 |
