# Supplementary material for: Low injury incidence and excellent return to sport after injuries in beach handball—a cross-sectional survey of 651 athletes
Source: BMC Sports Sci Med Rehabil. 2025 Aug 4;17:224. doi: 10.1186/s13102-025-01252-w (PMC12323119; doi:10.1186/s13102-025-01252-w)
Supplement: Supplementary file 8 — Additional file 8. Injury mechanism of acute injuries. [file 13102_2025_1252_MOESM8_ESM.docx]

| **Injury Mechanism of acute injuries** (multiple responses possible) | **Total number (n=102)** | **Percentage** |
| --- | --- | --- |
| **Contact** | **74** | **72.6** |
| Hit by an opponent coming down after a jump | 20 | 19.6 |
| Collision with an opponent (defense) | 17 | 16.7 |
| Collision with an opponent (offense) | 10 | 9.8 |
| Contact with the sand (excluding fall) | 8 | 7.8 |
| Contact with the ball | 6 | 5.9 |
| Hit by a team mate coming down after a jump | 5 | 4.9 |
| Collision with a team mate | 4 | 3.9 |
| Contact with the boundary line | 2 | 2.0 |
| Contact with the goal | 2 | 2.0 |
| Contact with out-of-bounds apparatus | 0 | 0.0 |
| **Jump** | **29** | **28.4** |
| Coming down on the sand | 23 | 22.5 |
| Coming down on an opponent after a jump | 5 | 4.9 |
| Coming down on a team mate after a jump | 1 | 1.0 |
| **Playing the ball** | **21** | **20.6** |
| Whilst throwing a 360°/spin shot | 10 | 9.8 |
| Whilst throwing 1 point shot, shooting specialist shot | 5 | 4.9 |
| Whilst blocking | 4 | 3.9 |
| Whilst passing | 1 | 1.0 |
| Whilst throwing an inflight shot | 1 | 1.0 |
| **Others** | **13** | **12.8** |
| Falling | 7 | 6.9 |
| Rapid change of direction | 4 | 3.9 |
| Rotation around planted foot | 2 | 2.0 |
| Acute overwork | 0 | 0.0 |
| Unintentionally hit by ball | 0 | 0.0 |
