# Supplementary material for: Low injury incidence and excellent return to sport after injuries in beach handball—a cross-sectional survey of 651 athletes
Source: BMC Sports Sci Med Rehabil. 2025 Aug 4;17:224. doi: 10.1186/s13102-025-01252-w (PMC12323119; doi:10.1186/s13102-025-01252-w)
Supplement: Supplementary file 9 — Additional file 9. Treatment after acute injuries. [file 13102_2025_1252_MOESM9_ESM.docx]

| **Treatment after acute injuries** (multiple responses possible) | **Total number (n=102)** | **Percentage** |
| --- | --- | --- |
| **Immediate treatment** | |  |
| “I carried on playing” | 30 | 29.4 |
| Ice therapy | 48 | 47.1 |
| Compression | 20 | 19.6 |
| Limb elevation | 12 | 11.8 |
| Immobilization with a splint | 8 | 7.8 |
| Hospital | 26 | 25.5 |
| “Nothing, I just stopped playing” | 18 | 17.6 |
| **Further treatment** | |  |
| Waited it out (missed training and competitions) | 66 | 64.7 |
| Regular painkillers | 24 | 23.5 |
| Anti-inflammatory medication | 29 | 28.4 |
| Injection/infiltrations | 5 | 4.9 |
| Physiotherapy | 60 | 58.8 |
| Stabilization in a brace | 20 | 19.6 |
| Immobilization in a splint | 9 | 8.8 |
| Immobilization in a cast | 6 | 5.9 |
| Surgery | 8 | 7.8 |
| Toe injury | 1 | 1.0 |
| Meniscus tear | 3 | 2.9 |
| Ligaments: Outer/ Lateral ankle ligament tear or bony avulsion | 1 | 1.0 |
| Joint: Dislocated shoulder | 1 | 1.0 |
| Head: Broken bone of the face or skull (other than nose) | 1 | 1.0 |
| Joint: Separated shoulder (acromioclavicular joint injury/dislocation) | 1 | 1.0 |
| **Treatment response** | |  |
| **Reduce stress** |  |  |
| Break from training/games/competitions | 65 | 63.7 |
| Reduction in training intensity | 17 | 16.7 |
| Reduction in game/competition intensity | 13 | 12.7 |
| Reduction in training frequency | 12 | 11.8 |
| Reduction in game/competition frequency | 9 | 8.8 |
| Reduction of intensity of training exercies especially affecting injured site | 11 | 10.8 |
| Reduction of physical activity in the workplace | 4 | 3.9 |
| **Regeneration** |  |  |
| Increased/more frequent breaks between training sessions | 4 | 3.9 |
| Increase in sleep | 8 | 7.8 |
| Dietary change or supplementation | 3 | 2.9 |
| **Modifications** |  |  |
| Modification of training exercises | 19 | 18.6 |
| Change in position | 2 | 2.0 |
| **Prevention** |  |  |
| Increase of stretching exercises | 11 | 10.8 |
| Increase of strength training exercises | 7 | 6.9 |
| Increase of proprioception/neuromuscular training exercises | 8 | 7.8 |
| **Medical** |  |  |
| Physiotherapy | 39 | 38.2 |
| Injection/infiltrations | 2 | 2.0 |
| Surgery | 8 | 7.8 |
| **Gear** |  |  |
| Brace | 11 | 10.8 |
| Taping | 25 | 24.5 |
| “I stopped playing permantly“ | 3 | 2.9 |
| “I carried on playing as usual and the injury resolved by itself” | 7 | 6.9 |
| Nothing applies | 5 | 4.9 |
