# Supplementary material for: Low injury incidence and excellent return to sport after injuries in beach handball—a cross-sectional survey of 651 athletes
Source: BMC Sports Sci Med Rehabil. 2025 Aug 4;17:224. doi: 10.1186/s13102-025-01252-w (PMC12323119; doi:10.1186/s13102-025-01252-w)
Supplement: Supplementary file 10 — Additional file 10. Treatment after overuse injuries. [file 13102_2025_1252_MOESM10_ESM.docx]

| **Treatment after overuse injuries** (multiple responses possible) | **Total number (n=72)** | **Percentage** |
| --- | --- | --- |
| **Reduce stress** |  |  |
| Break from training/games/competitions | 27 | 37.5 |
| Reduction in training intensity | 22 | 30.6 |
| Reduction in game/competition intensity | 11 | 15.3 |
| Reduction in training frequency | 17 | 23.6 |
| Reduction in game/competition frequency | 9 | 12.5 |
| Reduction of intensity of training exercies especially affecting injured site | 15 | 20.8 |
| Reduction of physical activity in the workplace | 3 | 4.2 |
| **Regeneration** |  |  |
| Increased/more frequent breaks between training sessions | 4 | 5.6 |
| Increase in sleep | 6 | 8.3 |
| Dietary change or supplementation | 4 | 5.6 |
| **Modifications** |  |  |
| Modification of training exercises | 14 | 19.4 |
| Change in position | 4 | 5.6 |
| **Prevention** |  | 0.0 |
| Increase of stretching exercises | 14 | 19.4 |
| Increase of strength training exercises | 17 | 23.6 |
| Increase of proprioception/neuromuscular training exercises | 8 | 11.1 |
| **Medical** |  | 0.0 |
| Physiotherapy | 29 | 40.3 |
| Injection/infiltrations | 6 | 8.3 |
| Surgery | 6 | 8.3 |
| (Chronic) spondylolisthesis (instability between vertebrae, possibly caused by hyperextension) | 2 | 2.8 |
| Tendon: Patella tendinopathy/ jumper’s knee | 1 | 1.4 |
| Knee injury, unspecified | 1 | 1.4 |
| Broken nose | 1 | 1.4 |
| Joint: Meniscus tear | 1 | 1.4 |
| **Gear** |  | 0.0 |
| Brace | 12 | 16.7 |
| Taping | 15 | 20.8 |
| “I stopped playing permantly“ | 2 | 2.8 |
| “I carried on playing as usual and the injury resolved by itself” | 4 | 5.6 |
| Nothing applies | 4 | 5.6 |
