# Supplementary material for: Low injury incidence and excellent return to sport after injuries in beach handball—a cross-sectional survey of 651 athletes
Source: BMC Sports Sci Med Rehabil. 2025 Aug 4;17:224. doi: 10.1186/s13102-025-01252-w (PMC12323119; doi:10.1186/s13102-025-01252-w)
Supplement: Supplementary file 11 — Additional file 11. Return to sport after acute injuries. [file 13102_2025_1252_MOESM11_ESM.docx]

| **Return to Sport after acute injuries** | **Total number (n=102)** | **Percentage** |
| --- | --- | --- |
| **“Have you returned to playing beach handball since your injury?”** |  |  |
| No | 7 | 6.9 |
| Yes | 95 | 93.1 |
| **“If no, what has kept you from going back?”** |  |  |
| Recent injury | 7 | 6.9 |
| **“How long did it take to return to light training/ activity (e.g. jogging, light training)?”** |  |  |
| “My injury did not make me stop light training / activity at any point” | 3 | 2.9 |
| Less than 1 week | 7 | 6.9 |
| 1 to 4 weeks | 52 | 51.0 |
| ~ 2 months | 20 | 19.6 |
| ~ 3 months | 7 | 6.9 |
| ~ 4 months | 1 | 1.0 |
| ~ 5 months | 1 | 1.0 |
| ~ 6 months | 1 | 1.0 |
| ~ 7 months | 1 | 1.0 |
| ~ 10 months | 1 | 1.0 |
| “I did not return yet, but I think I will” | 1 | 1.0 |
| Not applicable, injury recent | 7 | 6.9 |
| **“How long did it take to return to full beach handball training/ competition?”** |  |  |
| Less than a week | 0 | 0.0 |
| 1 to 4 weeks | 47 | 46.1 |
| ~ 2 months | 19 | 18.6 |
| ~ 3 months | 10 | 9.8 |
| ~ 4 months | 3 | 2.9 |
| ~ 5 months | 2 | 2.0 |
| ~ 6 months | 3 | 2.9 |
| ~ 8 months | 1 | 1.0 |
| ~ 9 months | 2 | 2.0 |
| ~ 12 months | 2 | 2.0 |
| > 1 year | 3 | 2.9 |
| I did not return yet, but I think I will | 3 | 2.9 |
| Not applicable, injury recent | 7 | 6.9 |
| **“How long did it take you to return to the physical level you were at before your injury?”** |  | 0.0 |
| “My injury did not make me perform beach handball at a lower level at any point” | 0 | 0.0 |
| Less than 1 week | 0 | 0.0 |
| 1 to 4 weeks | 38 | 37.3 |
| ~ 2 months | 13 | 12.7 |
| ~ 3 months | 11 | 10.8 |
| ~ 4 months | 5 | 4.9 |
| ~ 5 months | 2 | 2.0 |
| ~ 6 months | 6 | 5.9 |
| ~ 7 months | 1 | 1.0 |
| ~ 8 months | 1 | 1.0 |
| ~ 10 months | 1 | 1.0 |
| ~ 12 months | 4 | 3.9 |
| More than a year | 4 | 3.9 |
| “I did not return yet, but I think I will” | 2 | 2.0 |
| “I am still not at my previous level” | 7 | 6.9 |
| Not applicable, injury recent | 7 | 6.9 |
