# Supplementary material for: Low injury incidence and excellent return to sport after injuries in beach handball—a cross-sectional survey of 651 athletes
Source: BMC Sports Sci Med Rehabil. 2025 Aug 4;17:224. doi: 10.1186/s13102-025-01252-w (PMC12323119; doi:10.1186/s13102-025-01252-w)
Supplement: Supplementary file 12 — Additional file 12. Return to sport after overuse injuries. [file 13102_2025_1252_MOESM12_ESM.docx]

| **Return to sport after overuse injuries** | **Total number (n=72)** | **Percentage** |
| --- | --- | --- |
| **“Have you returned to playing beach handball since your injury?”** |  |  |
| No | 6 | 8.3 |
| Yes | 66 | 91.7 |
| **“How long did it take to return to light training/ activity? (e.g. jogging, light training)”** |  |  |
| “My injury did not make me stop light training / activity at any point” | 24 | 33.3 |
| Less than 1 week | 8 | 11.1 |
| 1 to 4 weeks | 17 | 23.6 |
| ~ 2 months | 7 | 9.7 |
| ~ 3 months | 2 | 2.8 |
| ~ 4 months | 2 | 2.8 |
| ~ 5 months | 1 | 1.4 |
| ~ 6 months | 4 | 5.6 |
| ~ 7 months |  | 0.0 |
| ~ 10 months | 1 | 1.4 |
| “I did not return yet, but I think I will” | 6 | 8.3 |
| **“How long did it take to return to full beach handball training/ competition?”** |  |  |
| “My injury did not make me stop light training / activity at any point” | 21 | 29.2 |
| Less than a week | 8 | 11.1 |
| 1 to 4 weeks | 17 | 23.6 |
| ~ 2 months | 7 | 9.7 |
| ~ 3 months | 7 | 9.7 |
| ~ 4 months | 2 | 2.8 |
| ~ 5 months | 3 | 4.2 |
| ~ 6 months | 1 | 1.4 |
| ~ 8 months | 1 | 1.4 |
| ~ 9 months | 2 | 2.8 |
| ~ 12 months | 1 | 1.4 |
| > 1 year | 4 | 5.6 |
| “I did not return yet, but I think I will” | 27 | 23.5 |
| **“How long did it take you to return to the physical level you were at before your injury?”** |  |  |
| “My injury did not make me perform beach handball at a lower level at any point” | 17 | 23.6 |
| Less than 1 week | 3 | 4.2 |
| 1 to 4 weeks | 14 | 19.4 |
| ~ 2 months | 6 | 8.3 |
| ~ 3 months | 4 | 5.6 |
| ~ 4 months | 3 | 4.2 |
| ~ 5 months | 1 | 1.4 |
| ~ 6 months | 1 | 1.4 |
| ~ 7 months | 1 | 1.4 |
| ~ 8 months | 1 | 1.4 |
| ~ 10 months | 2 | 2.8 |
| ~ 12 months | 2 | 2.8 |
| > 12 months | 2 | 2.8 |
| “I did not return yet, but I think I will” | 7 | 9.7 |
| “I am still not at my previous level” | 8 | 11.1 |
